# Protein Tyrosine Phosphatase CD45 As an Immunity Regulator and a Potential Effector of CAR-T therapy

**DOI:** 10.32607/actanaturae.25438

**Published:** 2023

**Authors:** D. V. Volkov, V. M. Stepanova, Y. P. Rubtsov, A. V. Stepanov, A. G. Gabibov

**Affiliations:** M.M. Shemyakin and Yu.A. Ovchinnikov Institute of Bioorganic Chemistry of the Russian Academy of Sciences, Moscow, 117997 Russian Federation

**Keywords:** CD45, T lymphocytes, B lymphocytes, T cell receptor, cancer, chimeric antigen receptor

## Abstract

The leukocyte common antigen CD45 is a receptor tyrosine phosphatase and one of the most prevalent antigens found on the surface of blood cells. CD45 plays a crucial role in the initial stages of signal transmission from receptors of various immune cell types. Immunodeficiency, autoimmune disorders, and oncological diseases are frequently caused by gene expression disorders and imbalances in CD45 isoforms. Despite extensive research into the structure and functions of CD45, the molecular mechanisms behind its role in transmitting signals from T-cell receptors and chimeric antigen receptors remain not fully understood. It is of utmost importance to comprehend the structural features of CD45 and its function in regulating immune system cell activation to study oncological diseases and the impact of CD45 on lymphocytes and T cells modified by chimeric antigen receptors.

## INTRODUCTION

**Fig. 1:**
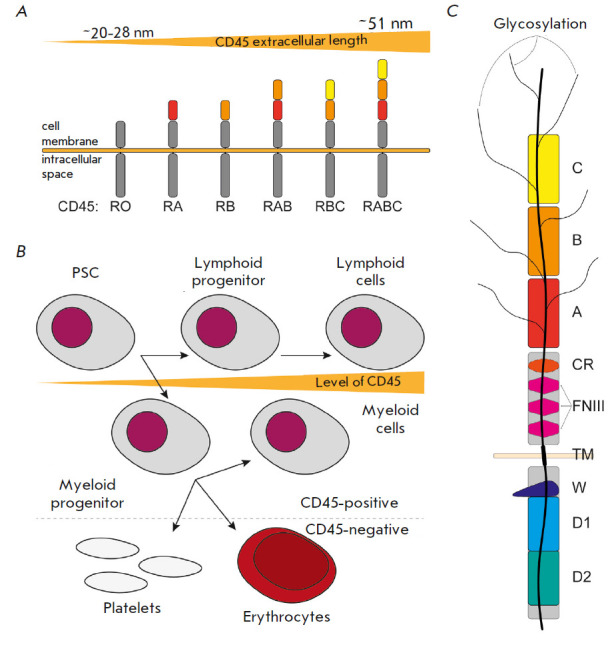
The structure and prevalence of CD45 isoforms in blood cells. (*A*) Six main CD45 isoforms found in humans differ in the composition of their extracellular part owing to the alternative splicing of pre-mRNA of the *PTPRC *gene; (*B*) CD45 resides on the membrane of all cells of hematopoietic origin except for platelets and erythrocytes. The amount of CD45 increases during cell differentiation; (*C*) the structure of CD45RABC. PSC – pluripotent stem cell; A, B, C – the extracellular domains of CD45 responsible for a particular isoform; CR – cysteine-rich region; FNIII – fibronectin type III domains; TM – transmembrane domain; W – wedge domain; D1 – domain with phosphatase activity; D2 – domain required for CD45 to function in the cell

Human protein tyrosine phosphatase (PTP) CD45 is encoded by the *PTPRC *gene containing 35 exons that have been characterized, and three of those (exons 4–6) [1, 2] contain homologous enhancers and silencers of alternative pre-mRNA splicing. Despite the theoretically expected significant versatility of possible variants, only six CD45 isoforms have been identified in humans: RO (exons 3, 7, and 8), RA (exons 3, 4, 7, and 8), RB (exons 3, 5, 7, and 8), RAB (exons 3, 4, 5, 7, and 8), RBC (exons 3, 5–8), and RABC (exons 3–8) (*Fig. 1A*). CD45 isoforms are found on all cells of hematopoietic lineage (except for anucleate erythrocytes and platelets); the level of CD45 correlates with the degree of cell differentiation [3, 4] (*Fig. 1B*).

The extracellular part of CD45 consists of five structural regions. The N-terminal region is long and heavily glycosylated. This very region is responsible for what receptor isoform it is. The remaining regions of the extracellular CD45 domain common to all the isoforms are the three fibronectin type III domains and the region carrying five conserved cysteine residues. Importantly, CD45 isoform regulates the sensitivity of T cells to activation upon antigen recognition. It is assumed that due to its bulky shape and structural rigidity, CD45 is expelled from the central region as the immune synapsis (IS) is formed and as the membranes of antigen-presenting cells (APCs) and T cells approach each other [5]. The number and type of CD45 isoforms in T cells vary depending on their differentiation degree: large CD45 isoforms are predominantly found in naïve and dormant cells. In turn, activated T cells synthesize CD45 isoforms in which either most or all the domains encoded by variable exons are absent [2]. The CD45 glycoprotein contains a single transmembrane domain (*Fig. 1C*) and three intracellular ones: the wedge domain D1, and D2. Frederick and colleagues demonstrated that only the proximal domain D1 exhibits phosphatase activity (previously, it was found that D2 is required for phosphatase functioning) [6, 7].

## CD45 FUNCTIONS IN IMMUNE CELLS

**The role and functions of CD45 in T cells**

**Fig. 2:**
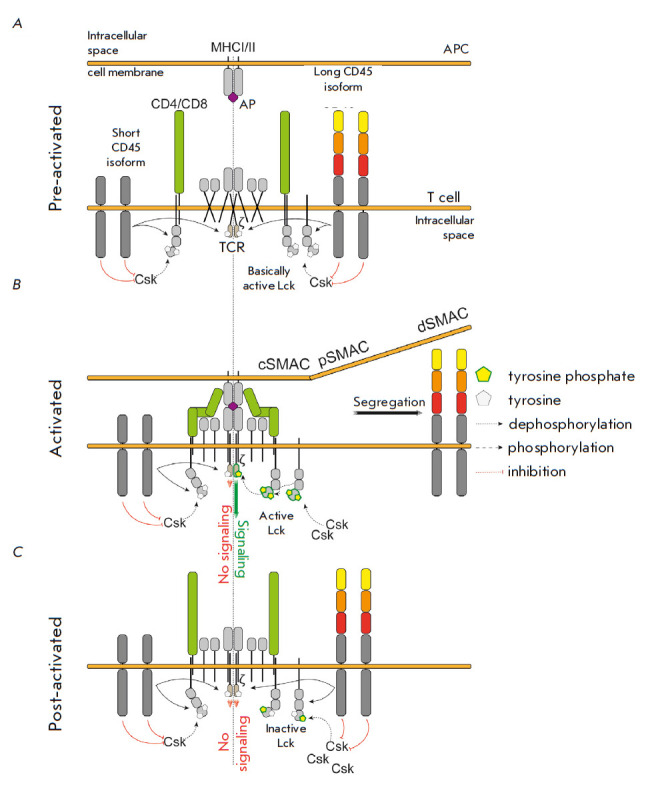
The role of CD45 in transmitting T-cell receptor activation signal. T cell activation stages are shown: the pre-active (*A*), active (*B*), and post-active (*C*) ones. During the activation cycle, the composition and phosphorylation of IS participants changes: first (*A*), Lck kinase exists in the state of basal activity due to dephosphorylation by CD45 phosphatase prevailing over phosphorylation by Csk kinase; in the active state (*B*), CD45 is “segregated” in dSMAC due to its rigid and bulky structure (long isoforms) and Lck becomes active due to autophosphorylation and prevalence over Csk and phosphorylates CD3ζ, which enables further signaling; short isoforms of CD45 gradually penetrating cSMAC are synthesized at this time, and Csk accumulates there, leading to a transition of Lck to its inactive form and the end of signaling (*C*). MHCI/II I – major histocompatibility complex class I or II; APC – antigen-presenting cell; PA – presenting antigen; ζ – CD3ζ; TCR – T-cell receptor; Lck, Csk – protein kinases; cSMAC, pSMAC, dSMAC – the central, peripheral, and distal supramolecular activation clusters, respectively

The involvement of CD45 in the activation of immune cells was first demonstrated for the T-cell receptor (TCR) signaling pathway. An analysis of T cells lacking CD45 expression showed that this phosphatase is essential at the initial stage of signal transduction from TCR [8] (*Fig. 2*). In a non-activated T cell, CD45 dephosphorylates protein tyrosine kinase (PTK) Lck and the CD3ζ subunit of the CD3/TCR receptor. Lck is the main substrate of CD45 phosphatase, which is capable of dephosphorylating both the inhibitory tyrosine residue (Y505) at the C-terminus of kinase and activating the tyrosine residue (Y394) [9, 10]. During dephosphorylation of the inhibitory tyrosine residue Y505, CD45 competes with tyrosine kinase Csk, which inhibits Lck [11]. Courtney and colleagues studied the dual function of phosphatase and inferred that CD45 regulates the intensity and frequency of the signal transduced via TCR by exerting an impact on different substrates. By varying the activity of CD45, they revealed that the phosphatase maintains a significant amount of Lck active but prevents the activation of CD3ζ. A detailed study of the dynamics of immune synapse formation showed that before activation, the TCR complex has a non-active conformation and does not interact with the major histocompatibility complex (MHC) class I or class II. Meanwhile, CD45 inhibits the recruitment of kinase Csk [12] and dephosphorylates CD3ζ and Lck (*Fig. 2A*). During cell interaction, CD45 and Lck molecules are first recruited to the central supramolecular activation cluster (cSMAC) via TCR. However, during IS formation, CD45 is expelled to the distal supramolecular activation cluster (dSMAC) [13, 14, 15, 16, 17] (*Fig. 2B*). The expulsion of CD45 from the IS is seemingly related to molecule size and the high level of glycosylation and sialation [18, 19, 20] (size reduction of the CD45 ectodomain increases colocalization of phosphatase and TCR, as well as reduces the activity of TCR [5, 20, 21, 22, 23]). Furthermore, CD45 needs to be removed from the IS center to shift the equilibrium in the central part of the synapse toward kinases. Changes in the balance enable the phosphorylation of CD3ζ, thus ensuring transduction of the signal for TCR activation. For the activation cycle to be completed, Csk molecules and the CD45RO isoform need to begin accumulating within the immune synapse [2]; the CD45RO isoform gradually penetrates into the central portion of the immune synapse and shifts the kinase–phosphatase equilibrium toward phosphatases, as well as dephosphorylates CD3ζ and activates the tyrosine residue in Lck (*Fig. 2C*). The signal intensity drops, and the composition of CD45 isoforms changes (toward increasing length and volume), while Lck returns to its basal activity state.

Presumably, through this mechanism for TCR regulation, CD45 impedes spontaneous T-cell activation, thus preventing hyperactivation and its negative sequelae [24] induced by low-affinity antigens or in the absence of an antigen. Zikherman and colleagues changed the levels of CD45 and Csk expression and showed that the balance between these molecules plays a crucial role in T-cell development. During the maturation of T cells in the thymus, the basal and inducible TCR signaling are regulated at the positive and negative selection stages. CD45 plays a positive and negative role simultaneously in antigen recognition. Variation of the Csk level regulates only the basal signal transduction. Meanwhile, an identical reduction in the Csk and CD45 levels leads to opposite changes in basal signaling by the same value. Therefore, a fluctuating CD45 level is needed for the following two processes to properly unfold: regulation of inducible signaling during positive and negative selection and compensation for Csk upon maintenance of the basal activity of T cells [25, 26]. In CD45-deficient mice with deleted exons 6 [27], 9 [28], or 12 [29], CD45 was shown to play a pivotal role in the transition of double negative (DN, CD4-CD8-) thymocytes to double positive (DP, CD4^+^CD8^+^) ones [30]. The initiation and gradual changes in the synthesis of CD4 and CD8 coreceptors during thymocyte differentiation depend on signals generated by pre- TCR and TCR. The involvement of CD45 is needed for these phenotypic and functional changes to occur. Deficiency of Lck, an important participant in TCR signaling controlled by CD45, manifested itself in a way similar to the lack of CD45 [31]: the transition of double-negative T cells to double positive ones in mice was disturbed [30], while the number of mature peripheral T cells was ≤ 5–10% of that for wild-type animals [27, 28].

**The role and functions of CD45 in B cells**

In B cells, CD45 also plays a crucial role in the modulation of the signal transduced via the B-cell receptor and is required to ensure normal B-cell development and an adequate response to an antigen. CD45-deficient mice were found to have defects in B-cell maturation [27, 28, 32]. Interestingly, the number of peripheral B cells does not decrease but their phenotype changes noticeably. The population of mature B cells in the spleen, as well as the population carrying the CD23 marker and MHC class II molecules, significantly decreases (IgDhigh IgMlow) [32]. In mice carrying a mutation within CD45 exon 9, the number of immature peripheral B cells is increased (IgMhigh) [28]. Importantly, CD45-deficient B cells did not proliferate in response to the stimulation of B-cell receptors (with polyclonal anti-IgD/anti-IgM antibodies); however, when other activation pathways were stimulated (by lipopolysaccharide (LPS), interleukin 4 (IL-4) and monoclonal anti-CD4 antibody), the proliferation of CD45-negative B cells was identical to that in control cells.

Cyster et al. [33] found that in response to antigen stimulation, naïve B cells isolated from CD45-deficient mice employed the ERK/RSK/EGR1 kinase pathways to a lesser extent and exhibited a low level of intracellular calcium mobilization. A difference in the markers of CD86 and CD54 activation was also observed: their level was lower in CD45-negative B cells compared to that in the CD45-positive ones. However, it was demonstrated by stimulating B cells with phorbol ester in combination with ionomycin that CD45- negative and CD45-positive B cells are activated identically.

It is essential to mention the role of CD45 in germinal centers and upon autoimmune diseases. It has been demonstrated that high-affinity autoreactive B cells fail selection in the bone marrow of native and CD45-deficient mice. However, the loss of CD45 expression allowed low-affinity autoreactive B cells to pass positive selection. Because of the lack of CD45, these B cells during selection did not induce the ERK/RSK/EGR1 pathway; intracellular calcium mobilization in response to the antigen in them was significantly lower compared to that in high-affinity cells, which provided protection to autoreactive cells against elimination. Therefore, CD45 regulates BCR and TCR activation in different ways. Unlike for TCR, the higher level of CD45 favorably affects BCR signaling, as well as it enhances the activation of the ERK/RSK/EGR1 and PI3K/AKT/mTOR kinase pathways and intracellular calcium mobilization [34]. In the case of increased CD45 expression, the Src family kinases (SFKs) involved in the BCR pathway are dephosphorylated at inhibitory tyrosine (Y507) more actively, whereas the level of phosphorylated activating tyrosine (Y416) in them remains unchanged [35]. Decreased CD45 expression has no effect on the Ca^2+^ level, since B cells contain CD148 phosphatase, which partially duplicates the functions of CD45 [35].

**The role and functions of CD45 in macrophages**

Leukocyte adhesion to the extracellular matrix and other cells is regulated by proteins belonging to the family of integrins [36, 37]. The targets of CD45 phosphatase activity, SFKs, are involved in the regulation of integrin-dependent phagocytosis, as well as macrophage differentiation and activation caused by adhesion [38, 39, 40]. Roach et al. have demonstrated that in the absence of CD45, the regulation of integrin-dependent adhesion is disturbed, while the activity of PTKs Hck and Lyn (SFKs exhibiting activity in myeloid cells) is increased [41]. The CD45-mediated regulation of Hck and Lyn kinases in macrophages differs from the regulation of SFKs in T and B cells, where CD45 activity is needed to a greater extent for the dephosphorylation of the inhibitory tyrosine residues at the C-termini of Lck and Fyn and the enhancement of their activity [42, 43, 44]. The simultaneously increased phosphorylation of C-terminal tyrosine residues and SFK activity in CD45-negative macrophages indicate that the phosphatase inhibits SFK. This possibly occurs due to the dephosphorylation of autocatalytic tyrosine.

**The role and function of CD45 in neutrophils**

Experiments on mice deficient in kinases Hck, Fgr, and Lyn [45] have demonstrated that a loss of SFK reduces neutrophil adhesion and the level of posttranslational modification of proteins. In those experiments, the Rab27a-dependent mobilization of neutrophil elastase and vesicles containing integrins α3β1 and α6β1 was out of balance. This also led to a disruption of the neutrophil migration through the vascular basement membrane and extravasation upon inflammation [45]. Hck and Fgr are involved in chemoattractant- dependent oxidative stress and F-actin polymerization [46]. SFKs also have something to do with the regulation of the mRNA transcription of many important cytokines and chemokines, which are synthesized by neutrophils either constitutively or upon stimulation (with interleukins-1, -6, -8, -10, -12; tumor necrosis factor-α (TNF-α); granulocyte– macrophage colony-stimulating factor, etc.) [47, 48]. Liles et al. [49] showed that CD45 activation by antibodies increases the level of oxidative stress induced by neutrophil activators. In turn, Harvath et al. [50] demonstrated that CD45 interacts with the molecules that are coupled to receptors for leukotriene B4 and the complement component C5a and that it regulates neutrophil chemotaxis in response to stimulation with the respective ligands. Gao et al. [51] revealed that colocalization of CD45 receptors and FcγRIIa reduces the antibody-dependent cytotoxicity of neutrophils, while simultaneously increasing IL-6 production upon FcγRIIa-mediated activation. Zhu et al. [52] showed that CD45 in neutrophils increases signaling of G-protein-coupled receptors and enhances Ca^2+^ mobilization and the activity of the PI3K and ERK kinases.

**The role and function of CD45 in dendritic cells**

Dendritic cells (DCs) play a crucial role in sustaining the relationship between the innate and adaptive immunity. CD45 is involved in the formation of these functional distinctions, since its specific isoforms (CD45RB) mark different DC populations. Foreign molecules bind to pattern recognition receptors (TLRs being among them), thus initiating the program of dendritic cell maturation. This process determines the further DC-mediated activation of naïve T cells together with their response to the presented antigen [53]. Early stages of TLR signaling pathways are believed to regulate SFKs [54]. The signal from TLRs increases translation of the co-stimulatory molecules that are required for activating naïve T cells and secretion of proinflammatory cytokines such as IL-12, IL-6, and TNF-α, which affect the type of generated effector T cells [55]. TLR is one of the key components of DC activation. Although the main elements of the signaling pathways of these receptors are known, the contribution of SFK has not been thoroughly described. A comparative analysis of dendritic cells isolated from native and CD45-deficient mice [56] showed that CD45 is not required for dendritic cell development but affects their maturation induced by TLR agonists. CD45 has an impact on the phosphorylation of Lyn, Hck, and Fyn and reduces LPSinduced activation of Lyn. CD45 had a favorable effect on TLR4-induced secretion of proinflammatory cytokines and interferon-β (IFN-β). Moreover, CD45 exhibited different effects on TLR activation: negative (TLR2 and TLR9 or MyD88-dependent cytokine production) and positive (TLR3 and TLR4 or MyD88- independent IFN-β secretion) [56].

**The role and function of CD45 in NK cells**

Many of the known NK cell receptors (CD16, NK1.1, NKG2D, NKp44, etc.) are associated with the intracellular proteins FcεRIγ, DAP10 or DAP12, which contain immunoreceptor tyrosine-based activation motifs (ITAMs) [57]. CD45 regulates the activity of SFKs, which phosphorylate tyrosine residues in ITAM and trigger the activation signaling pathways [58]. Hesslein and colleagues demonstrated that during the stimulation of the Ly49H and NKG2D receptors, CD45- negative NK cells are only 20% less toxic than control CD45-positive NK cells, while no differences are observed during the stimulation of the CD16 receptor. However, cytokine and chemokine secretion was much lower in CD45-negative NK cells in [59]. Therefore, CD45 can have different effects on the same activation signaling pathway. Signal intensity and/or duration are responsible for CD45 recruitment. Cytotoxic granules are released within several minutes after receptor activation, in direct proximity from many components of the cellular signaling pathways involved in activation. In turn, cytokine secretion [60] is a longer process that involves the transduction of the transcriptional activation signal, mRNA synthesis and maturation, as well as translation and secretion. Therefore, persistent signaling is needed for cytokine release, whereas short-term stimulation is sufficient for eliciting a cytotoxic effect in DCs [61].

**The role of CD45 in cancer**

In hematopoietic cancers, CD45 expression depends on the cancer type. Thus, Feuillard and colleagues found that in patients with chronic lymphocytic leukemia (CLL), atypical tumor cells and the low level of CD45 on their surface are positive markers of patient survival [62]. Loss of CD45 was detected in patients with Hodgkin lymphoma [63] and childhood acute lymphoblastic leukemia (ALL) [64]. The higher CD45 expression in patients with ALL is associated with a higher risk of tumor recurrence [65]. It still remains unclear how CD45 is involved in the pathogenesis of multiple myeloma (MM) [66]. Patients with MM simultaneously had both CD45-positive and CD45-negative tumor cells [67]. The increased CD45 expression enhances the sensitivity of MM cells to 17-dimethylaminoethylamino- 17-demethoxygeldanamycin, an inhibitor of HSP90 chaperone [67], and various apoptotic stimuli (e.g., oxidative stress and endoplasmic reticulum stress) [68]. Regardless of whether MM cells contained CD45, stimulation with IL-6 was able to unlock the JAK/STAT signaling pathways; however, only CD45-positive cells can proliferate after activation [69]. The overall survival chances of patients with the predominance of CD45-positive MM cells was lower compared to that for patients with a predominance of CD45-negative cells [70]. On the other hand, the role of CD45 has been characterized much better in patients with diffuse large B cell lymphoma (DLBCL). Phosphatase CD45 acts as a galectin-3 receptor [71]; transcription of the gene encoding it is upregulated in DLBCL cells [72]. Galectin-3 exhibits antiapoptotic activity [73]. When binding to CD45, galectin-3 remains anchored to the cell membrane. Its removal was shown to increase the number of apoptotic tumor cells [71].

The immunosuppressive tumor microenvironment (TME) is a determinant factor of the resistance of solid tumors to immunity. Several layers comprising various types of cells can be differentiated within the tumor microenvironment; an appreciably significant portion of these cells are myeloid-derived immune cells that become immunosuppressive under tumor signaling (myeloid-derived suppressor cells, MDSC) [74, 75]. MDSCs typically positively express the surface markers CD11b and Gr-1. The key function of these cells is suppression of the effector functions of NK and T cells [76, 77]. MDSCs were also shown to potentiate the immunosuppressive activity of APCs [78], which may inhibit the activity of T cells. van Vliet et al. demonstrated that macrophages and DCs inhibit effector T cells through MGL (macrophage galactose-type lectin), one of the C-type lectin receptors [79]. The interaction between MGL and CD45 of effector T cells reduced their proliferation and caused apoptosis. Schuette et al. [80] also revealed that the mannose receptor on DCs interacts with CD45 on cytotoxic T cells, thus resulting in their inhibition, reprogramming, and development of immunological tolerance.

## THE EFFECT OF CD45 ON THE ACTIVITY OF CARS

Chimeric antigen receptors (CARs) are recombinant receptors that allow targeting immune cells to surface tumor-associated antigens (TAAs) [81]. CAR is a transmembrane molecule comprising an antigen recognition domain (it typically is a single-chain variable antibody fragment), the transmembrane domain, intracellular costimulatory domains (CD28, 4-1BB, and OX40 being most common), and the signaling domain (typically, CD3ζ) [82]. Several generations of CARs are currently known; they differ in the number of costimulatory domains or a set of auxiliary intracellular domains [83]. Despite the similar functionality, the effects of any activation of CARs and TCRs on cell proliferation and the cytotoxic response are different [84].

**Structural and functional distinctions between TCRs and CARs**

Unlike TCRs, which activate T cells after the recognition of 1–10 MHC molecules, several thousand surface TAA molecules are needed for CAR activation [84, 85]. There are many distinctions between CARs and TCRs, which are responsible for the increased threshold of the antigen content required for efficient activation of T cells. First, it is the receptor/ligand affinity: TCRs ensure MHC–antigen binding with micromolar affinity [85], while CARs bind their ligands with nanomolar affinity [86]. The increased affinity of CAR binding alters the kinetics that turn off the receptor, as well as its ability to be repeatedly activated and its mechanoreceptor function; these properties are believed to contribute to the ability of TCRs to perceive low ligand levels [87, 88]. After interaction with the antigen, the TCR and CD3δ, CD3ε, CD3γ, and CD3ζ bound to it assemble multicomponent signaling complexes [89, 90]. CARs interact with some signaling proteins of TCR; however, the quantitative and qualitative changes in the assembly of signaling complexes and the structure of IS alters the sensitivity to antigens [84, 91]. Imaging of the CAR and TCR synapses revealed that CAR synapses depend on the interaction between intercellular adhesion molecule-1 and integrin αLβ2 to a lesser extent, and that they are characterized by altered Lck localization compared to that for TCR [92, 93, 94]. Actin rings in the CAR synapse are much smaller than those in the TCR synapse, thus causing faster transduction of mechanical signals and dissociation of a CAR T cell from the target cell. Signal in the CAR synapse is initiated faster and is more intense, while signal duration is shorter than in the TCR synapse. This fact accelerates the involvement of the CAR T cell in the interaction with the target cell, causes fast release of cytotoxic granules in the IS, and rapid cytolysis of tumor cells [94].

**The effect of CD45 on signal transduction upon activation of CARs**

**Fig. 3:**
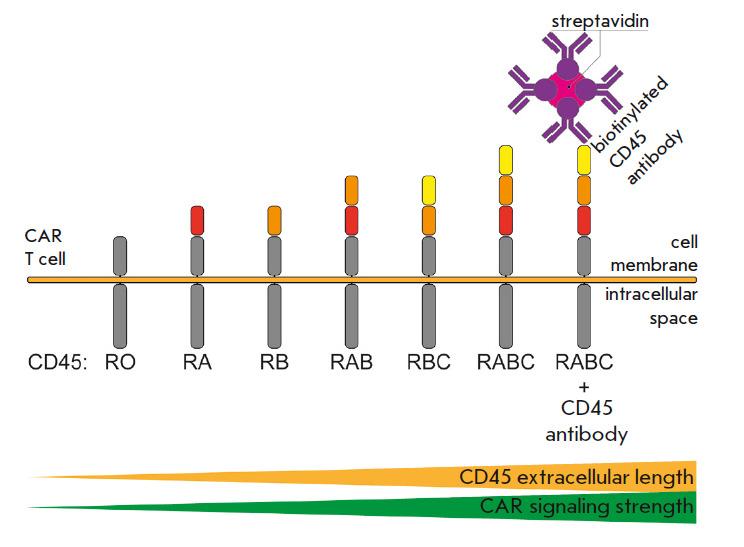
The length of the extracellular part of CD45 influences CAR signaling. CD45 segregation from the immune synapse and CAR signaling strength as a result of an increase in the length of the extracellular portion of phosphatase. CAR T cell – T cell modified by the chimer - ic antigen receptor

The release of CD45 molecules into the distal region of the immune synapse facilitates the phosphorylation of Lck and CD3ζ and it ensures tight contact in the receptor–antigen complex [5]. Karlsson et al. demonstrated that CD45 segregation is also required for activation of CAR19 to proceed, which is similar to TCR activation [95]. It is logical that activation of both CAR and TCR depends on CD45 segregation from the domain where the immune synapse takes shape. In both cases, CD45 substrates (SFKs) are expected to play a crucial role in signal transduction. The effects of CAR size, the distance to the TAA epitope to be recognized, and the CD45 length on the transduction of signals from CAR and activation of CAR19 T cells have been identified [96]: an increased size of the extracellular domain of CAR reduces CD45 segregation from the immune synapse area, phosphorylation of the signaling participants, and release of proinflammatory cytokines. The same dependence is also observed upon varying the distance to the TAA epitope to be recognized. An increase in CD45 length has an opposite effect regardless of the reason why it happens: this is true both for different CD45 isoforms and for the case when the molecule volume is increased by using specific antibodies (*Fig. 3*). These findings support the kinetic segregation model for CAR T cells [97] proposed by Karlsson et al. in their experiments [95].

## CONCLUSIONS

Disruption of phosphorylation is one of the many causes of cancer. CD45 and other phosphatases play a positive role in oncogenesis by regulating the pro-oncogenic mechanisms; therefore, they are potential candidates for a targeted elimination of tumors or for increasing their sensitivity to chemo- or radiation therapy. Progress continues to be made in the research into CD45 using novel methods for designing and screening CD45 inhibitors, as well as technologies that enable synthesis inhibition and allow one to change the composition of CD45 isoforms in human hemocytoblast (CRISPR/Cas9). The known CD45 ligands (pUL11, E3/49K) and their analogs are also considered promising targets for cancer therapy. The importance of CD45 synthesis by tumor cells in predicting the clinical outcome of patients with CLL, ALL, MM, and DLBCL has been demonstrated. The course of many oncological diseases may also depend on the activity of this phosphatase. An important feature of CD45 is the typical composition of isoforms, which depends on cell differentiation. T cells with a naïve phenotype express CD45RA, while central memory and effector memory T cells express the CD45RO isoforms only. This segregation allows one to easily isolate the T-cell population of interest and then obtain CAR T cells with tailored properties. For example, memory T cells not expressing the CD45RA marker can be used to reduce the risk of graft-versus- host disease [98, 99]. The abundance of phosphatase among lymphoid and myeloid cells, as well as the high receptor level on the membrane [4], makes CD45 an extremely attractive target for CAR-T therapy both for hematopoietic tumors and upon the conditioning of recipient’s hematopoiesis preceding bone marrow transplantation. Along with the other methods used to control the activity of CAR T cells, the idea of regulating CAR activation by changing the CD45 length is very promising [100, 101]. Recent developments indicate that when designing a novel CAR, one needs to take into account the ratio between the sizes of the chimeric antigen receptor, the targeted antigen, and CD45 [102]. CD45 knockout is another performance potential associated with the enhancement of the CAR T cell. CD45 is essential for T-cell development and maturation; however, the absence of CD45 expression on CAR T cells can increase the safety of adoptive immunotherapy by reducing the risk of adverse reactions related to TCR signaling.
